# Metagenomic data of DNA viruses of poultry affected with respiratory tract infection

**DOI:** 10.1016/j.dib.2017.11.033

**Published:** 2017-11-13

**Authors:** Manisha R. Sajnani, D. Sudarsanam, Ramesh J. Pandit, Tejas Oza, Ankit T. Hinsu, Subhash J. Jakhesara, Siddhardha Solosanc, Chaitanya G. Joshi, Vaibhav D. Bhatt

**Affiliations:** a Bharathiar University, Coimbatore, Tamil Nadu, India; b Department of advanced Zoology and Biotechnology, Medical Lab-technology & Bio medical instrumentation, Loyola College, Chennai, India; c Department of Animal Biotechnology, Anand Agricultural University, Anand, Gujarat, India; d Department of Pharmaceutical Sciences, Saurashtra University, Rajkot, Gujarat, India; e Institute of Medical Microbiology, Madras Medical College, Chennai, India

**Keywords:** DNA Virome, Metagenomics, Next generation sequencing

## Abstract

The incidence and severity of respiratory diseases in commercial broiler chicken flocks have increased recently in India because of intensification of the broiler industry. Viral population are predominant in respiratory tract infections and they pose continuous economic burden to poultry industry by causing severe economic losses through decreased productivity [1], [2]. To understand viral metagenome of poultry associated with respiratory infections, we performed DNA virome sequencing and data analysis of broilers from 8 districts of Gujarat State in India. We report high quality sequencing reads and highly abundant DNA viral population present in the infected broiler birds. The raw sequencing data used to perform metagenomic analysis is available in the Sequence Read Archive (SRA) under the BioProject No. PRJNA322592 and Accession No. MAUZ00000000, MAVA00000000, MAVB00000000, MAVC00000000, MAVD00000000, MAVE00000000, MAVF00000000, MAVG00000000 (https://www.ncbi.nlm.nih.gov/bioproject/?term=PRJNA322592).

**Specification Table**

**Table:** 

Subject Area	Biology
More specific subject area	Metagenomics
Type of data	DNA viruses metagenomic data
How data was acquired Sequencer Machine	Shotgun DNA sequencing using Ion Torrent PGM platform
Data format	Raw data in fastq files
Experimental factors	Total DNA was extracted from tracheal and nasal swab from infected broilers
Experimental features	Whole genome shotgun sequencing and population dynamics of DNA viral metagenome of birds affected with respiratory tract infection
Data source location	Districts of Anand in Gujarat state, India (22.5645°N, 72.9289°E)
Data accessibility	Data is submitted to NCBI on SRA submission portal with BioProject PRJNA322592 and it is in the public repository. The direct URL to data is https://www.ncbi.nlm.nih.gov/bioproject/?term=PRJNA322592.

**Value of data**

• The reported data is first ever approach to determine the whole DNA virome associated with respiratory infections in the poultry.
• Data will help the researchers to identify novel functional genes, microbial pathways, antibiotic resistance genes, interactions and co-evolution between microbiota and host i.e. infected broilers.
• Data will allow global detection of known and unknown viruses associated with respiratory tract infection in poultry [1], [2].
• Data can be used for designing effective preventive measures and develop vaccines for poultry diseases.

## 1 Data

Data presented here contains information about i) sample collection from 8 poultry farms located in the Gujarat State; ii) sample wise nanodrop readings of extracted DNA sample; iii) sample wise quality filtering of raw reads to remove reads with pred score <20 and read length <50 using PrinSeq tool; iv) Sample wise host specific screening against the Gallus gallus genome to fetch unmapped or unaligned reads for the downstream analysis; v) The *de novo* assembly report from SPAdes assembler for merged reads generated from 8 samples and vi) reference guided mapping of merged 8 sample reads against the virus database using map to read approach of CLC Genomic Workbench to identify highly abundant viruses present in the broiler chicken.

## 2 Experimental design, materials and methods

### 2.1 Sample collection

In the present work, tracheal and nasal swab of 34 broilers affected with respiratory diseases were collected in sterile tubes from 8 different poultry farms located in Gujarat, India (Supplementary file 1). The collected samples were then filtered through 0.2 μm filter and filtrates were immediately stored at −80 °C. For purifying viruses associated with tracheal tissue, the tracheal tissue was processed via blending into a ~20% homogenate in sterile phosphate buffered saline (PBS) followed by centrifugation at 7500 rpm (5500G) centrifugation steps for 15 min at 4 °C. A stepwise filtration process involving 0.8 μm and 0.45 μm was used to remove eukaryotic and bacterial cells and nuclei. Virus-sized particles were pelleted by ultracentrifugation for 5 h. at 4 °C (113,000G) using CsCl density gradient centrifugation.

### 2.2 DNA isolation

DNA was extracted from each samples separately using extraction kit from Roche. Before processing DNA samples were pooled in equimolar concentration for each farm. The samples were amplified with whole genome amplification kit from Qiagen for DNA. The qualitative and quantitative evaluation of extracted DNA was done using nanodrop 1000 UV–vis spectrophotomer as well as 0.7% agarose gel electrophoresis (Supplementary file 2). For isolating DNA viruses, after homogenisation samples was treated by DNase I for removal of host background DNA. Viral DNA was extracted using standard phenol:chloroform extraction method and amplified to increase quantity.

### 2.3 Library preparation and next generation sequencing

Amplified products were used further for library preparation as manufacture's protocol. In brief, the samples were sonicated to generate fragments of 400–500 bp size range followed by end polishing and adaptor ligation to the free ends. The quality and average size of the library were accessed on the Agilent 2100 bioanalyzer with the DNA high sensitivity kit (Agilent Technologies, USA). Genomic libraries were clonally amplified, enriched and subjected to sequencing run using Ion Torrent PGM 316 Chip with 300 bp chemistry following the manufacturer's protocol. Individual farms samples were separated using molecular barcoding. Data were transferred to high end cluster having 2 TB RAM and 100 nodes for further analysis.

### 2.4 Quality filtering of data and host specific screening

Raw reads were scanned with pred score >20 and read length >50 bp for quality filtering using PrinSeq tool (http://prinseq.sourceforge.net/) (supplementary file 3). Host specific screening was performed by mapping against host genome of *Galus galus* from NCBI (ftp://ftp.ncbi.nlm.nih.gov/genomes/Gallus_gallus/) and assembly name: Gallus_gallus-5.0) using Bowtie2.2.8 (https://sourceforge.net/projects/bowtie-bio/files/bowtie2/2.2.8/) with default parameter (Supplementary file 4). The same procedure was followed for each samples separately. Reads that mapped partially or completely with host genomic sequences were removed from further analysis. Unmapped reads (unaligned to host sequences) were considered as clean reads and used for assembly and downstream analysis.

### 2.5 *De novo* assembly of DNA viromes

Assembly for each sample was performed separately using high quality reads using SPAdes assembler (http://bioinf.spbau.ru/en/spades3.7) based on multiple k-mer (k-mer length 21, 33, and 55). The best assembly was obtained at 55 k-mer size was used for downstream analysis. The detailed assembly statistics having number of contigs, total contig bases, N50 size, and GC% is provided in the supplementary file 5.

### 2.6 Predominant viruses

Highly abundant viruses were predicted by using reference mapping approach on the high quality reads using virus genome database from NCBI (ftp://ftp.ncbi.nlm.nih.gov/genomes/Viruses/). We used “map to read” CLC genomics workbench version 7.0.4.1 for predicting predominantly occurring viruses associated with the infection. The mapped reads showing >90% coverage are provided in Table 1.

**Table 1:** Highly abundant viruses obtained by reference mapping using CLC Genomic Workbench.

**Accesion No.**	**Viruses name**	**Mapped reads**	**% Genome covered**
NC_015396.1	Avian gyrovirus 2, complete genome	2357	100
NC_018401.1	Gyrovirus 4, complete genome	987	100
NC_001427.1	Chicken anemia virus, complete genome	21,923	99.91
NC_022789.1	Gyrovirus Tu789, complete genome	124	98.86
NC_015630.1	Human gyrovirus type 1, complete genome	3225	97.62
NC_026808.1	Mongoose feces-associated gemycircularvirus b strain 160b, complete genome	10,993	92.42
