# Supplementary material for: Metagenomic data of DNA viruses of poultry affected with respiratory tract infection
Source: Data Brief. 2017 Nov 13;16:157–60. doi: 10.1016/j.dib.2017.11.033 (PMC5847339; doi:10.1016/j.dib.2017.11.033)
Supplement: Supplementary file 1 — Supplementary material [file mmc1.docx]

**Conflict of interests:**

None of the authors have any financial or personal relationships that could inappropriately influence or bias the content of the data presented here. The authors declare that they have no conflict of interest.
